# Engineering magnetic nano-manipulators for boosting cancer immunotherapy

**DOI:** 10.1186/s12951-022-01760-8

**Published:** 2022-12-31

**Authors:** Bin Yan, Siyao Wang, Chen Liu, Nana Wen, Hugang Li, Yihan Zhang, Hao Wang, Ziyi Xi, Yi Lv, Haiming Fan, Xiaoli Liu

**Affiliations:** 1 Laboratory of Resource Biology and Biotechnology in Western China, Ministry of Education, Provincial Key Laboratory of Biotechnology of Shaanxi Province, Northwest University, Xi’an, 710069 Shaanxi China; 2 College of Chemistry & Materials Science, Northwest University, Xi’an, 710127 Shaanxi China; 3 Institute of Regenerative and Reconstructive Medicine, Med-X Institute, First Affiliated Hospital of Xi’an Jiaotong University, Xi’an, 710049 Shaanxi China; 4 National Local Joint Engineering Research Center for Precision Surgery & Regenerative Medicine, Shaanxi Provincial Center for Regenerative Medicine and Surgical Engineering, First Affiliated Hospital of Xi’an Jiaotong University, Xi’an, 710061 Shaanxi China

**Keywords:** Magnetic nanoparticles, Magnetic hyperthermia, Immunogenicity, Immune-checkpoint blockade therapy, Nanodelivery system

## Abstract

Cancer immunotherapy has shown promising therapeutic results in the clinic, albeit only in a limited number of cancer types, and its efficacy remains less than satisfactory. Nanoparticle-based approaches have been shown to increase the response to immunotherapies to address this limitation. In particular, magnetic nanoparticles (MNPs) as a powerful manipulator are an appealing option for comprehensively regulating the immune system in vivo due to their unique magnetically responsive properties and high biocompatibility. This review focuses on assessing the potential applications of MNPs in enhancing tumor accumulation of immunotherapeutic agents and immunogenicity, improving immune cell infiltration, and creating an immunotherapy-sensitive environment. We summarize recent progress in the application of MNP-based manipulators to augment the efficacy of immunotherapy, by MNPs and their multiple magnetically responsive effects under different types of external magnetic field. Furthermore, we highlight the mechanisms underlying the promotion of antitumor immunity, including magnetically actuated delivery and controlled release of immunotherapeutic agents, tracking and visualization of immune response in real time, and magnetic regulation of innate/adaptive immune cells. Finally, we consider perspectives and challenges in MNP-based immunotherapy.

## Introduction

Immunity is the most important weapon for host defense against pathogens and gene mutations [1–3]. Since William Coley’s observation in 1893 that *Streptococcus pyogenes* infection caused tumor regression in sarcoma patients [4], various immunotherapies have been used to treat tumors; to date, several different types of immunotherapy have been approved for clinical use against a variety of tumors. Tumor immunotherapy represents a notable landmark in modern medicine [5].

The principle of cancer immunotherapy is based on modulation and activation of the immune system to recognize and eradicate malignant cells. Immunotherapy can elicit immune memory that provides long-term protection against tumor recurrence. To date, various immunotherapies, including immune checkpoint inhibitors, adoptive T cell therapy, cancer vaccines, and oncolytic viruses, have achieved progress in clinical studies. Notably, immune checkpoint blockade therapy has been approved by the U.S. Food and Drug Administration for first/second-line treatment of various solid tumors [6]. However, despite these advances, most immunotherapies remain less than satisfactory, with high failure rates in late-stage clinical trials [7] mostly due to low immunogenicity, weak antigen presentation, minimal T cell infiltration, and high expression of inhibitory receptors and cytokines [8, 9]. These characteristics allow immune escape of tumor cells from immune cells, hindering the production of a sufficiently strong tumor-specific immune response. In addition, most immunotherapeutic agents cannot be effectively delivered into the tumor due to poor water solubility, easy degradation, or non-specific targeting [10–12], which largely limit their antitumor efficacy.

The past 20 years have witnessed a remarkable increase in studies focused on inorganic nanomaterials owing to their unique physicochemical properties attributed to their reduced dimensions [13, 14]. Recent advances in inorganic nanomaterials have contributed to the development of nano-based drug delivery systems [15], allowed monitoring of immunological effects in real time and immunological regulation of the tumor microenvironment (TME), and, subsequently, the improvement of therapeutic efficacy [16–18]. In particular, focus has been given to enhancing the efficacy of antitumor immunotherapy by exploiting magnetic nanoparticles (MNPs) and their magnetically responsive effects under an external magnetic field, which can greatly improve the delivery efficiency of nanomedicine systems. Prominent examples of applications of MNPs in immunotherapy research include: (1) effective loading, remote magnetic field-controlled delivery, and on-demand release of immunotherapeutic agents [19–22]; (2) real-time tracking of the dynamic process of the immune response by magnetic resonance imaging (MRI) [19, 23]; (3) regulating the immunological TME by producing tumor-killing hydroxyl radicals, which is based on the nanozyme activity of MNPs [24, 25]; (4) MNP-mediated magnetic force to modulate cell function and fate, such as disrupting the cell membrane or cytoskeleton, leading to apoptosis and the release of tumor-associated antigens (TAAs) [26]; and (5) MNP-mediated magnetic hyperthermia to induce immunogenic tumor cell death, to promote macrophage polarization to a pro-inflammatory M1 phenotype, and to accelerate T lymphocyte infiltration into the tumor site [27–29]. Compared to other inorganic nanomaterials, MNPs can also simultaneously achieve efficient in vivo MRI tracking of the immune response and immunological modulation through magneto-physicochemical effects, which can temporally optimize the duration, dose, and schedule of treatment [30]. The development of both MNPs and magnetic biomedical engineering provides the possibility of solving intractable problems in immunotherapy, such as enhancing immunogenicity and lessening immune suppression to fulfil therapeutic effects in vivo. However, due to our poor understanding of both MNPs and their magnetically responsive effects in vivo, progress and new advances in this field have been slow, leading to limited clinical application.

**Fig. 1:**
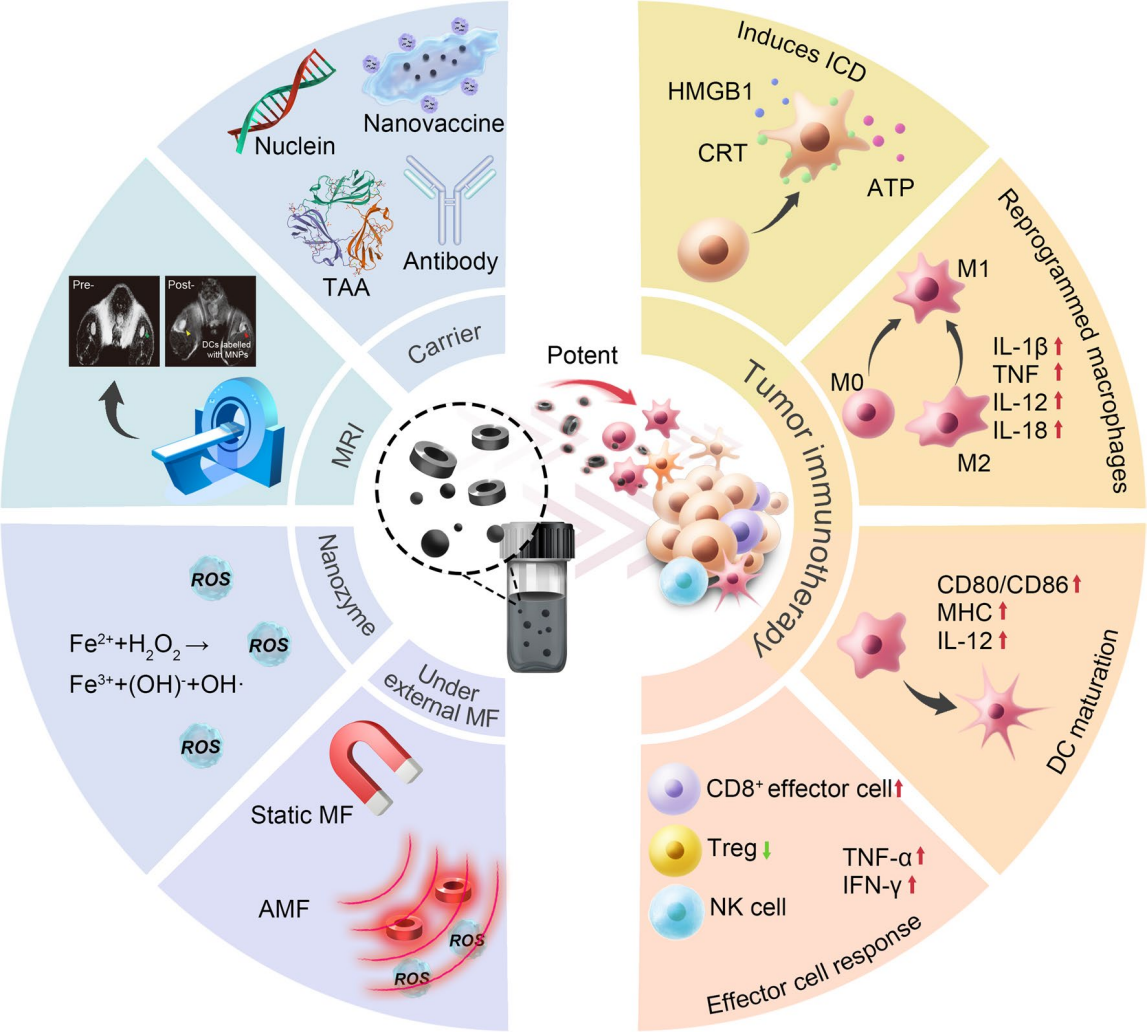
Schematic showing MNP-based manipulators for potent tumor immunotherapy. Applications of MNPs in immunotherapy research include: effective loading of immunotherapeutic agents; real-time tracking of the dynamic process of the immune response by MRI; remote magnetic field-controlled delivery of immunotherapeutic agents; and MNP-mediated magnetic hyperthermia to regulate immunological TME, e.g., to induce immunogenic tumor cell death, to promote macrophage polarization to a pro-inflammatory M1 phenotype, and to accelerate T lymphocyte infiltration into the tumor site. MF: magnetic field, including static magnetic field and alternating magnetic field (AMF)

Herein, we summarize the contribution of MNPs and their multiple magnetically responsive effects under different types of magnetic field to the advancement of immunotherapy. We focus on utilization of the distinct functions of MNPs in promoting antitumor immunity; applications include magnetically actuated delivery and controlled release of cargo, tracking and visualization of immune response in real time, magnetic regulation of innate/adaptive immune cells, and magnetic activation of antitumor immunity (Fig. 1). We will also summarize opportunities and challenges in MNP-based immunotherapy.

## MNP-based magnetically controlled delivery of immunotherapeutic agents

Effective delivery of immunotherapeutic agents into the tumor site for action is a principal goal of cancer immunotherapy. Currently, tumor immunotherapy depends on immune adjuvants [31–33], antibodies [34–37], or inhibitors [38–41] to activate immunity or block immunoprevention checkpoints to enhance antitumor responses. However, immunotherapeutic agents do not always work and have shown only moderate success, attributed mostly to limitations in solubility and targeting that prevent enrichment at the tumor site, an immunosuppressive microenvironment that causes unstable responsiveness to immune agents, and resistance and side effects associated with multiple dosing. To address these issues, MNPs may be developed to exhibit a versatile nanoscale surface for functionalization with biomacromolecules and NPs. Additionally, magnetically activated cargo delivery could be achieved by an external magnetic field pulling MNPs through tissue, offering a remote and non-invasive approach for tumor targeting. Moreover, tailoring the biological interface of MNPs loaded with immunotherapeutic agents to molecules with good biocompatibility and strong interaction with receptors overexpressed by tumor cells [42, 43] can improve specificity, reduce non-specific phagocytosis, and enhance accumulation in tumors. Additionally, the easy fabrication, low toxic effects, and high stability of MNPs as carriers also make them attractive in antitumor immunotherapy. MNPs have the ability to provide excellent contrast-enhanced MR imaging signals, allowing the monitoring and prediction of immunotherapeutic efficacy, and permitting tailoring for individualized treatment and precision medicine [44]. The immune response in vivo is a complex and dynamic interaction process among multiple cell types. The optimum dose rate and frequency of administration of immunotherapeutic agents will depend upon the physiological status of the patient during treatment, such as their general physical condition and their responsiveness to a given dosage. MNP-based MR imaging can be used to monitor treatment and adjust dosage and/or timing.

### Delivery of immune adjuvants by MNPs

Immunotherapy restores the human immune system and reactivates the anti-tumor immune cycle to control and inhibit tumor growth, metastasis, and recurrence. However, the tumor immunosuppressive microenvironment largely hinders antitumor immune responses, thereby resulting in low immunotherapeutic efficacy. Immune adjuvants act as immunomodulators enhancing immunogenicity and sustaining long-term immune responses, either specifically or non-specifically, and preceding or during co-administration with antigens, all of which have received increasing attention in recent years [45]. Immune adjuvants work directly on immune cells to promote their proliferation and differentiation, thereby inducing a more powerful immune response [46]. Currently, there are five FDA-approved immune adjuvants already in clinical use but their efficiency in activating immune responses leaves room for improvement. Because of their short half-life, easy catabolism, poor cellular uptake, and rapid degradation and elimination, immune adjuvants are administered in multiple and high doses, leading to adverse side effects, increased drug resistance, and poor patient compliance. Thus, it is vital to design optimal delivery systems for carrying and dispensing the hydrophobic immune adjuvants, particularly unstable or easily degradable molecules, to the appropriate cells of the immune system, thus benefiting the generation of a robust and durable immunological response.

Cytosine–phosphate–guanine (CpG) oligodeoxynucleotides are common immune adjuvants in cancer therapy, and act as Toll-like receptor 9 (TLR9) agonists to trigger T helper 1 (Th1) cell-mediated immune activation, involving maturation, proliferation, and differentiation of various type of immune cells. MNPs with controllable size and surface properties possess the advantage of having a ‘suitable’ vehicle size, which make them particularly effective vehicles for the delivery of immune adjuvants. TLR agonist CpG oligodeoxynucleotides delivered by MNPs provoked a robust immune response against cancer cells. For example, a pathogen-mimicking magnetic MnO NPs system was used to carry foreign DNA (unmethylated CpG) [47], and was able to enter human head and neck squamous cell carcinoma cells in vitro, selectively targeting and activating the TLR9 pathway. Due to their MR imaging capabilities, the cellular trafficking and transportation of CpG-carrying MnO NPs could be monitored. Furthermore, an Fe_3_O_4_ NP-based CpG delivery system showed better bioactivity and enhanced cellular uptake relative to CpG [48]. Compared to free CpG, delivering CpG by Fe_3_O_4_ NPs resulted in a substantial antitumor efficacy by stimulating adaptive immune responses in C26 colon cancer and 4T1 breast cancer xenograft models in vivo. An Fe_3_O_4_ NP-based vaccine with an optimized size of 40 nm was developed to directly deliver ovalbumin antigen (OVA) and TLR9 agonists to endosomal TLR9 in dendritic cells (DCs) in lymph nodes [49]. It was demonstrated that this micro-dosed magnetic nanodelivery system greatly enhanced adaptive immune responses in vivo, providing protection against various types of tumor challenge (*e.g.*, gastric cancer, melanoma, breast cancer) [50–54].

Based on the unique characteristics of MNPs, which can respond to a magnetic field, MNPs also have the ability to control the movement and concentration of the delivered agents in the targeted tissue under the influence of an external magnetic field, permitting MNPs to be used as carriers of immunotherapeutic agents with enhanced delivery efficiency. For example, MNPs functionalized with CpG were internalized into endosomal compartments of N9 microglia cells, and, upon exposure to a magnetic field, the movement of MNP-CpG-loaded microglia could be controlled with an external magnetic field, which established the possibility of using an MNP both to deliver an immunostimulatory cargo to cells and to control its trafficking [55].

In comparison with the numerous studies on the delivery of chemotherapeutics, little research has been conducted on magnetically controlled delivery of immunotherapeutic agents. Magnetic targeting should be extendable to immune cells by labeling desired cell populations with MNPs, or by optimally coating the MNPs to further enhance immune cell activation and delivery efficiency.

### Delivery of TAAs by MNPs

DCs are specialized antigen-presenting cells (APCs) that have the potential to induce antitumor immunological responses [56]. DC-based therapeutic vaccines have been explored for many years, by administration of autologous monocyte-derived DCs that have been loaded ex vivo with different kinds of antigens [57]. Therapeutic immunity was shown in preliminary results to occur only in a very small fraction of tumor cases using ex vivo-generated antigen-bearing DCs [58, 59]. DC-based immunotherapy has various challenges: first, sufficient antigens must be effectively delivered to DCs to stimulate the generation of specific cytotoxic T lymphocytes (CTLs) and tumor killing [60, 61]. Second, the migration must be tracked in vivo [62, 63]. Biocompatible MNPs, such as Fe_3_O_4_ NPs, are excellent choices for the delivery of antigens into DCs due to their large surface area, which allows multiple agents to be loaded, and their intrinsic detectability by MRI [64]. Furthermore, they are commonly recognized as the most clinically translatable and multifunctional materials for biomedicine. MNPs can act as tumor vaccines by delivering tumor antigens into the patient’s body, and induce cellular and humoral immune responses to control or destroy tumors [65, 66].

**Fig. 2:**
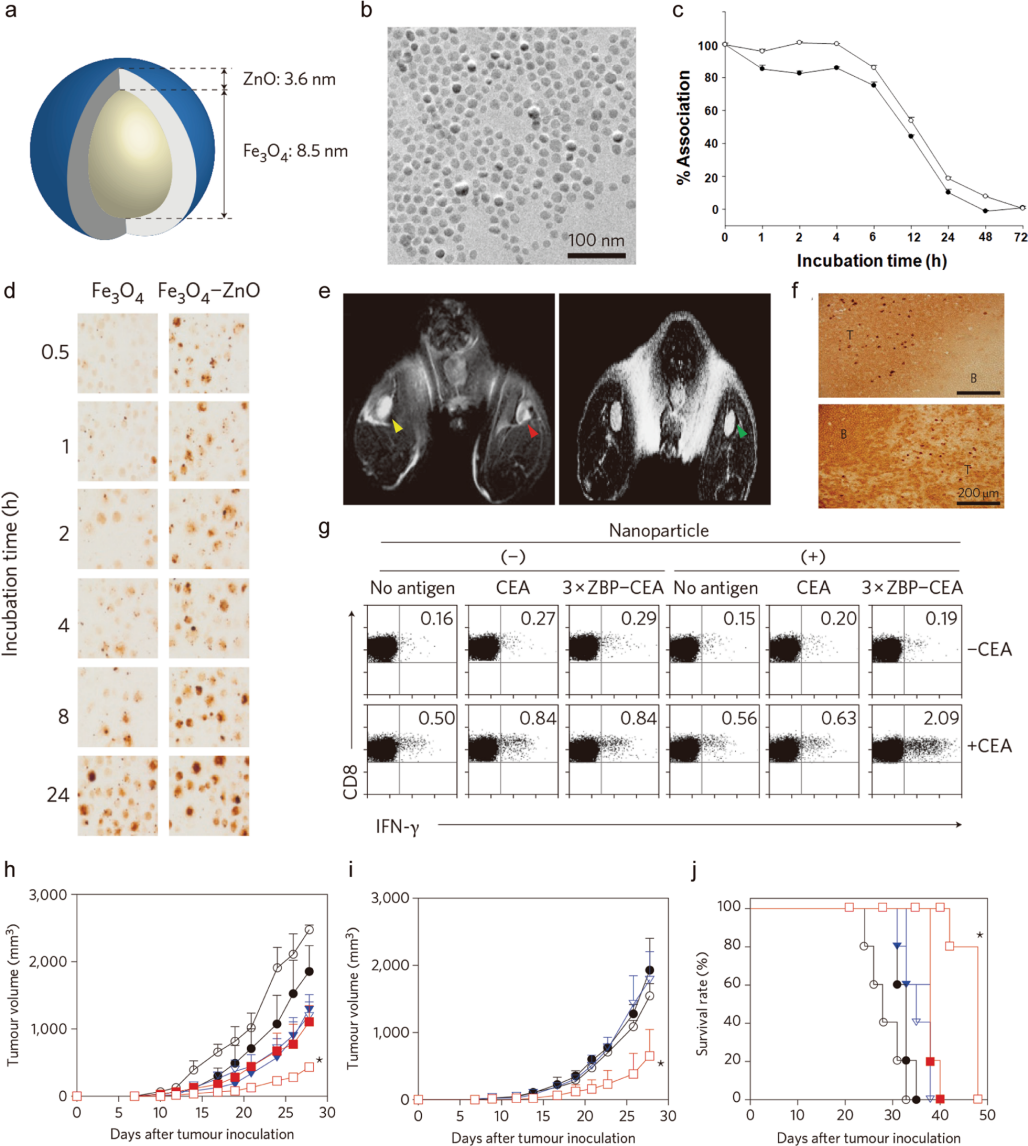
**a** Diagram of a core–shell NP. **b** TEM image of monodispersed spherical particles. **c** Time course of ZnO-binding peptide association with NPs in cell culture medium. **d** Intracellular NPs visualized by DAB-enhanced Prussian blue staining of DCs labeled with 100 mg/mL Fe_3_O_4_ NPs or Fe_3_O_4_–ZnO NPs after incubation for the indicated time. **e** In vivo MRI images of (left) draining lymph nodes of a mouse injected with DCs labeled with Fe_3_O_4_–ZnO (red arrowhead) or ZnO NPs (yellow arrowhead) into the ipsilateral footpads. Right shows a draining lymph node (green arrowhead) of a mouse injected with cell-free Fe_3_O_4_–ZnO NPs. **f** Representative immunohistochemistry of draining lymph nodes after injection with Fe_3_O_4_–ZnO NP-labeled DCs (dark brown dots). T, T cell zone (Thy1.2^+^); B, B cell follicle (B220^+^). **g** CEA-specific, IFN-γ^+^ CD8^+^ T cell responses of mice immunized with DCs. **h, i** Tumor volume of C57BL/6 mice (**h**) and CEA–transgenic C57BL/6 mice (**i**) injected with MC38/CEA cells. **j** Survival rate of mice injected with MC38/CEA cells. Data are expressed as means ± SD; *P < 0.05 [67]. Copyright 2011, Springer Nature

A core–shell MNP (iron oxide–zinc oxide) with a size of 15.7 nm (Fig. 2a, b) was used to deliver carcinoembryonic antigen (CEA) to DCs ex vivo [67]. More than 95% of DCs took up a large number of MNPs after only 1 h of incubation even without surface modification or using any transfection agents (Fig. 2c, d). Additionally, these MNPs also acted as a visualizing agent to monitor MNP-labeled DC trafficking by MRI in vivo after injection into the hind footpads of C57BL/6 mice (Fig. 2e). MNP-labeled DCs were mainly located in the central Thy1.2^+^ T cell region but not in B220^+^ B cell follicles (Fig. 2f). There were no changes in phenotype or viability after combining MNP-loaded DCs with a TAA. Thus, MNP-labeled DCs induced an intense anti-CEA immune reaction even in hosts with immune tolerance (Fig. 2g). In comparison with control groups, tumor growth was dramatically inhibited in both C57BL/6 mice (Fig. 2h) and transgenic C57BL/6 mice spontaneously expressing human CEA (Fig. 2i) following immunization with MNP-labeled DCs. All mice survived at day 40 only after treatment with MNP-labeled DCs (Fig. 2j).

Another example of the use of MNPs as an antigen delivery vehicle to DCs was reported by the Chung [68] group. Through the use of MNPs, the ability of DCs to capture OVA antigens was enhanced. Following internalization of OVA–MNPs, the migration and homing of DCs into lymph nodes was monitored using MR imaging. Thus, DC-based immunotherapies using MNP-delivered antigens for the stimulation and tracking of DCs in vivo could activate the anti-tumor immunological response and ensure consistent clinical efficacy.

**Fig. 3:**
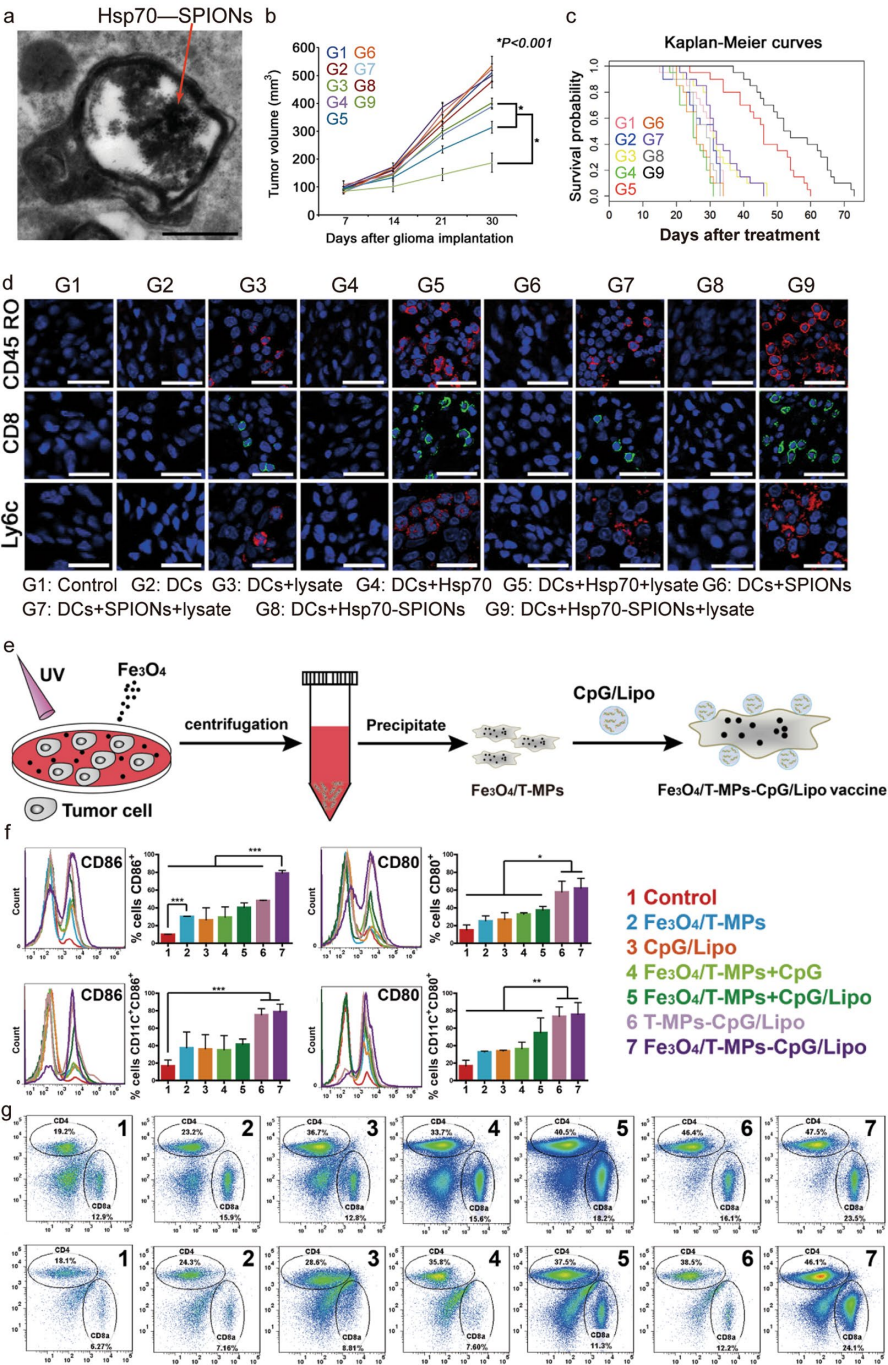
**a** TEM image of DCs co-incubated with Hsp70–SPIONs; scale bar = 200 nm. **b** Tumor volume in different experimental groups. **c** Kaplan–Meier survival curves for one control and eight experimental groups. **d** Immunofluorescence staining of glioma cryosections for Ly6c^+^ (red) NK cells, CD45RO^+^ (red) and CD8^+^ (green) T cells; scale bar = 25 μm [69]. Copyright 2015, Elsevier. **e** Nano–Fe_3_O_4_-carrying tumor-derived antigenic microparticles (T-MPs) surface-decorated with CpG-loaded liposomes to yield an anticancer vaccine (Fe_3_O_4_/T-MP–CpG/Lipo). **f** Fe_3_O_4_/T-MP–CpG/Lipo induced the up-regulation of CD86, CD80, and CD40 on macrophages (upper) and bone marrow-derived DCs (lower). Statistical significance was calculated by one-way ANOVA with Bonferroni’s post-test; *P < 0.05, **P < 0.01, ***P < 0.001. **g** Analysis of CD3^+^CD4^+^ CD3^+^CD8^+^ T cells in the lymph nodes (upper) and spleen (lower) [70]. Copyright 2019, American Chemical Society

Nanovaccines based on superparamagnetic iron oxide nanoparticles (SPIONs) offer a new way to evoke a systemic immune response against tumors. The ability of SPIONs to activate the immune system increased after they were bonded with heat shock protein 70 (Hsp70) [69], which is considered as an antigenic peptide chaperone. Hsp70–SPIONs stimulate tumor-specific CD8^+^ cytotoxic T cell responses by transmitting immunopeptides from tumor lysates to DCs (Fig. 3a). Administration of DCs loaded with Hsp70–SPIONs and tumor lysates into C6 glioma rats led to the suppression of tumor development and prolongation of survival (Fig. 3b, c). Meanwhile, interferon (IFN)-γ levels increased in serum, and the infiltration of memory CD45RO^+^ and cytotoxic CD8^+^ T cells inside the tumor was enhanced (Fig. 3d). Similarly, nano-Fe_3_O_4_ was used to convert tumor-derived antigenic microparticles (T-MPs) into cancer vaccines (Fe_3_O_4_/T-MPs) loaded with CpG to obtain a vaccine (Fe_3_O_4_/T-MPs–CpG/Lipo) (Fig. 3e) [70]. This vaccine was shown to elicit a powerful tumor antigen-specific immune response through APCs. Moreover, vaccines within the TME could re-educate tumor-associated macrophages (TAMs) to the M1 phenotype via the uptake of nano-Fe_3_O_4_ (Fig. 3f), inducing massive CTL infiltration and transforming ‘cold’ tumors into ‘hot’ tumors (Fig. 3g).

### Delivery of immune checkpoint-blocking antibodies by MNPs

Immune checkpoint proteins are immunosuppressive molecules that are commonly expressed on the surface of tumor cells or immune cells, and are involved in regulating the balance between T cell/TAM activation and tolerance [71], with proven clinical benefits [72]. Antibodies targeting cytotoxic T lymphocyte-associated protein 4 (CTLA-4) [73–75], or blocking the programmed death-1–programmed death-ligand 1 (PD-1/PD-L1) axis [76–78], as well as CD47 monoclonal antibodies (mAbs) that inhibit the interaction between CD47 and signal-regulatory protein-α (SIRPα) [79–82], are immune checkpoint-blocking drugs approved to treat various solid and hematologic cancers. Among these, the overexpression of CTLA-4 and/or PD-1/PD-L1 often restricts the activity of CTLs and allows tumors to evade immune surveillance and killing by the immune system. Blocking the PD-1/PD-L1 axis has shown unexpected clinical benefits compared to anti-CTLA-4 therapy in response rate, survival, and toxic effects, making anti-PD-1/PD-L1 agents first-line immunotherapeutics [83]. However, the response rate of PD-1/PD-L1 checkpoint inhibitors is less than 30%, and immunotoxicity/autoimmunity compromise the therapeutic response.

**Fig. 4:**
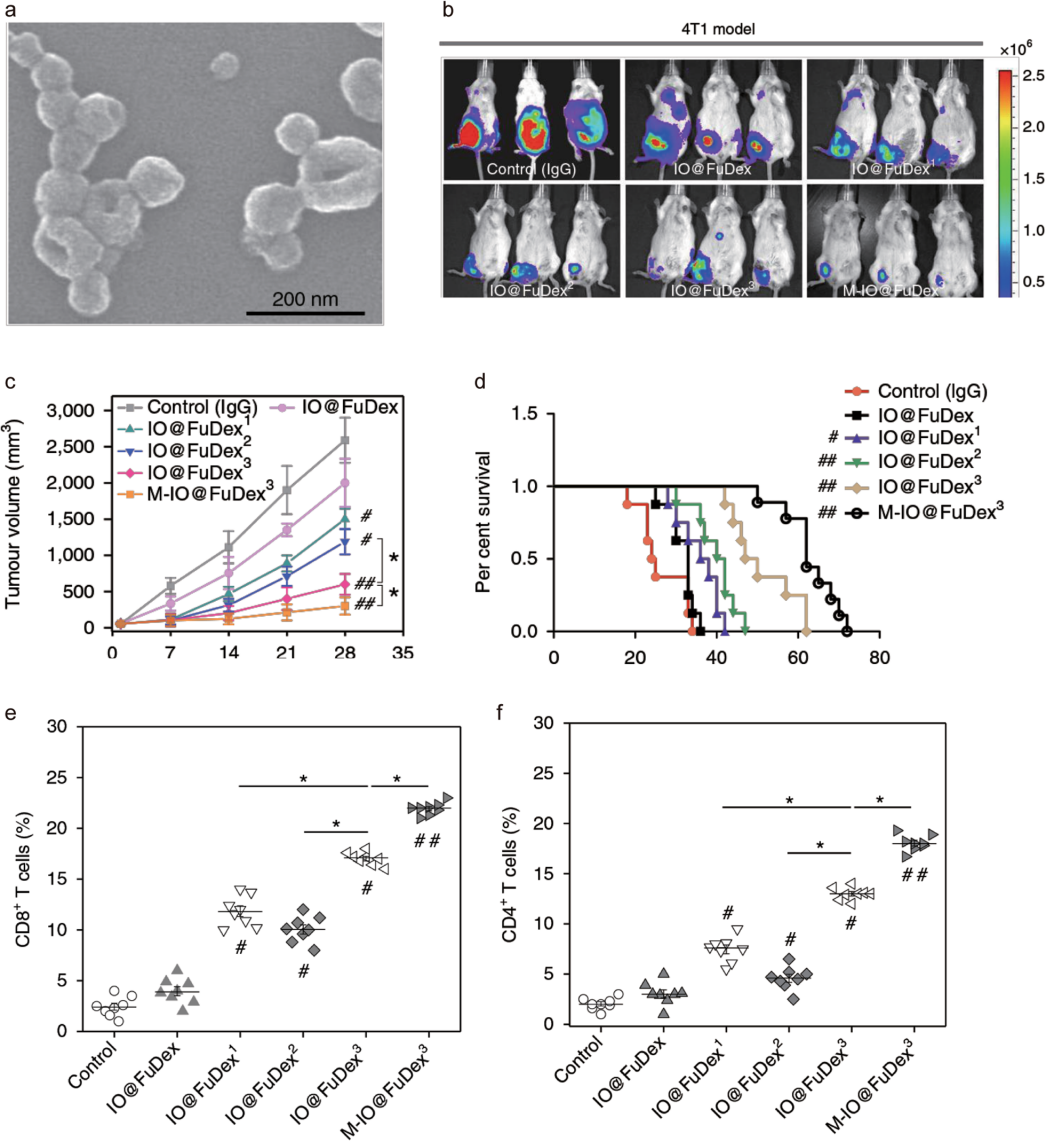
**a** SEM of IO@FuDex^3^. **b** IVIS images of the growth of luciferase-expressing 4T1 tumors at 4 weeks after first treatment with IO@FuDex^3^ formulations. **c**, **d** Tumor volumes and survival curves of mice receiving treatment with different IO@FuDex^3^ formulations. **e**, **f** Significant increase in CD8^+^ and CD4^+^ T cells after treatment with IO@FuDex^3^ formulations. Data in c, d are expressed as mean ± SD, n = 8 biologically independent animals. ^#^P < 0.05 and ^##^P < 0.01 compared with the control group (IgG); *P < 0.05 and **P < 0.01 compared between groups using a paired two-way Student’s *t*-test [85]. Copyright 2018, Springer Nature

MNPs are commonly used as carriers for the enrichment of immune checkpoint inhibitors within the tumor site as well as to combine these with MNP-induced physicochemical effects, enhancing both the immune response and antitumor efficacy. For example, an MNP-based TME-responsive nanocarrier was used to deliver and release a short D-peptide antagonist of PD-L1 (named P^D^PPA-1), which could block the immune checkpoint to favor the modulation of the immunosuppressive TME and the activation of CTLs, achieving effective treatment [84]. A fucoidan–dextran-based magnetic nanoagent (IO@FuDex^3^) (Fig. 4a) was developed, which labeled a checkpoint inhibitor (anti-PD-L1) and T cell activators (anti-CD3 and anti-CD28) [85]. The IO@FuDex^3^ exhibited magnetophoresis in response to a magnetic field, facilitating the active enrichment of immune cargos at the tumor site via magnetic control and minimizing off-target effects. Compared to their soluble form, an enhanced delivery efficiency of immunological agents was achieved by in vivo magnetic manipulation using IO@FuDex^3^. This enabled the reinvigoration of tumor-infiltrating lymphocytes and repair of the immunosuppressive TME, consequently improving the therapeutic efficacy and extending the median survival in a subcutaneous triple-negative breast 4T1 cancer xenograft tumor model (Fig. 4b–d). Additionally, IO@FuDex^3^ plus magnetic force driving induced significantly enhanced numbers for both CD8^+^ in CD3^+^ and CD4^+^ in CD3^+^ T cells, as well as reduced regulatory T cell numbers within the tumors (Fig. 4e, f), all of which improvements arose from the enhanced delivery efficiency of cargos by MNP-based manipulation.

CD47 mAbs suppressed the CD47–SIRPα axis, thus activating TAMs to phagocytose tumor cells. Monitoring of the CD47 mAb therapeutic response longitudinally in vivo through minimally invasive imaging methods needs to be developed. Based on the findings that ferumoxytol NPs (an FDA-approved iron supplement) could be phagocytosed by TAMs and detected by MR imaging in mouse models and patients, using CD47 mAbs to block the CD47–SIRPα interaction induced phagocytosis of both NPs and tumor cells, resulting in dramatically improved r_2_ relaxivities and increased numbers of M1 macrophages [86]. Thus, this could be used for MRI monitoring of the response of macrophages to CD47 mAb immunotherapy and could be applied to clinical trials with CD47 mAbs.

## Intrinsic immunotherapeutic activity of MNPs

Fe_3_O_4_ NPs have the unique advantage of acting as a generator of hydroxyl radical (OH·) reactive oxygen species (ROS) by the Fenton reaction in TME or within cancer cells [87]. The resulting toxic ROS can promote an immune response and antitumor immunotherapeutic effects, such as the induction of immunogenic cell death (ICD), the mediation of macrophage polarization, and the enhancement of T cell infiltration [88], as well as being effective tumor cell killers through disruption of redox balance [89–92].

### Fe-based MNPs for ICD induction

**Fig. 5:**
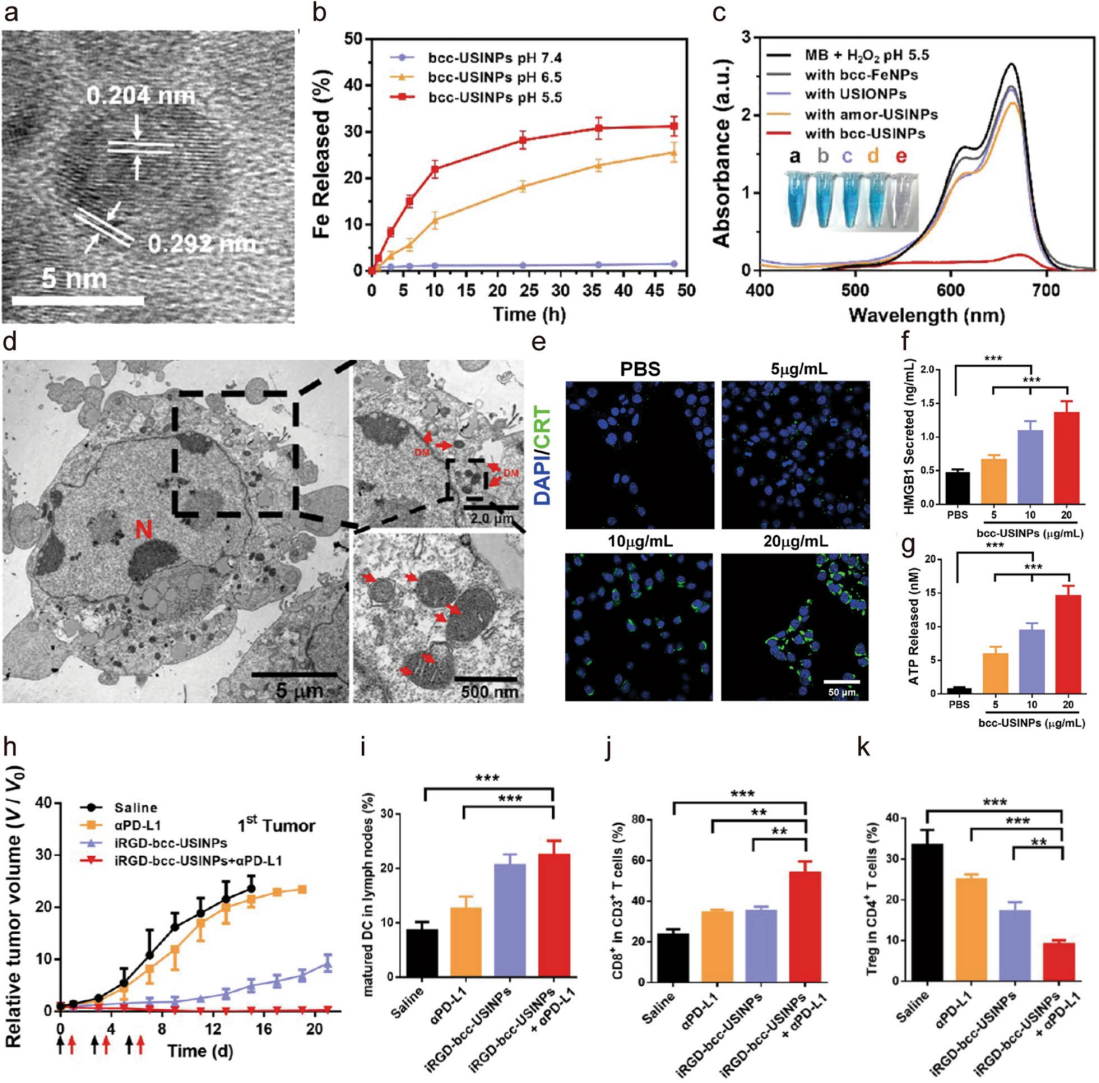
**a** High-resolution TEM of bcc-USINPs. **b** Cumulative Fe release from bcc-USINPs. **c** UV/visible absorption spectra of methylene blue solution in pH 5.5 buffer containing 1 mM H_2_O_2_ after adding bcc-Fe NPs, USIONPs, amor-USINPs, and bcc-USINPs to a concentration of 50 μg/mL [Fe]. **d** Bio-TEM image of a cross section of a HepG2 cell. Arrows indicate damaged mitochondria. **e** Fluorescence microscopy images of CRT expression on the MC38 tumor cell surface after treatment with PBS or bcc-USINPs. **f**, **g** HMGB1 secreted and ATP released from MC38 cells. **h** Average growth curves of MC38 tumor-bearing C57BL/6 mice after various treatments. **i** DC maturation in lymph nodes of MC38 tumor-bearing mice (gated on CD11c^+^ MHC II^+^ DC cells) after various treatments for assessment by flow cytometry. **j** FCM examination of the intratumor infiltration of CD8^+^ T cells (gated on CD3^+^ T cells). **k** Regulatory T (Treg) cell (gated on CD4^+^ T cells) frequencies in tumors after different treatments. Data are expressed as means ± SD (n = 3); **P < 0.01 and ***P < 0.001 [94]. Copyright 2021, American Chemical Society

Fe-based MNPs have been demonstrated to induce ferroptosis via the Fenton reaction and acceleration of ROS for cancer-specific therapy. Ferroptotic damage is accompanied by the release of damage-associated molecular patterns (DAMPs), such as the migration of calreticulin (CRT) on the cell surface, the secretion of adenosine triphosphate (ATP), and the release of high mobility group protein B1 (HMGB1), to further stimulate the immune system to enhance the immunotherapy [93, 94]. Fe-based MNPs can induce ICD to enhance DC maturation and activate adaptive T cell responses. Furthermore, Fe-based MNP-induced ICD can potentiate immunotherapy. The efficiency of ICD induction is largely dependent on the efficacy of ROS production. For example, ultrasmall single-crystal Fe NPs (bcc-USINPs) with a 2 nm Fe (0) core and a ~ 0.7 nm Fe_3_O_4_ shell (Fig. 5a) were stable in the physiological environment but highly unstable in the acidic TME, because the Fe_3_O_4_ shell was selectively etched by acid and the Fe (0) core was exposed (Fig. 5b) [94]. Benefiting from a highly reactive Fe (0) core in a H_2_O_2_-overproducing and acidic TME (Fig. 5c), the bcc-USINPs could effectively induce ferroptosis, as demonstrated by the regulation of widely used markers of ferroptosis, *e.g.,* the down-regulation of glutathione peroxidase 4 (GPX4) and ferroptosis suppressor protein 1 (FSP1), and the up-regulation of AcylCoA synthetase long-chain family member 4 (ACSL4). Furthermore, based on this, the bcc-USINPs induced ICD of tumor cells at extremely low concentrations (Fig. 5d–g). In contrast, CRT exposure was undetectable on the surface of tumor cells after treatment with unstable bcc-Fe NPs, ultrasmall Fe_3_O_4_ NPs, or ultrasmall amorphous Fe NPs at concentrations the same as, or even higher than, those of bcc-USINPs. Furthermore, following combination with anti-PD-L1 antibodies, bcc-USINP-induced ferroptosis significantly increased the antitumor immune response and produced a powerful immune memory (Fig. 5h–k).

### MNPs reprogram TAMs within the TME

TAMs are a vital target for tumor immunotherapy. TAMs are the major leukocytes of the TME and have unique phenotype and function. M1-type macrophages release pro-inflammatory cytokines and present antigens, acting as immunosurveillance agents and promoting immune responses; M2-type macrophages secrete suppressive cytokines, have a weaker antigen-presenting ability, and down-regulate immune responses, while M1 and M2 macrophages regulate each other to maintain the TME. MNPs have been used to polarize M2 macrophages into M1 macrophages by regulating the macrophage phenotype, reversing the tumor immune microenvironment and improving the efficacy of subsequent immunotherapy, and levels of iron-related proteins among macrophage subtypes may be important to sustain the polarized state.

**Fig. 6:**
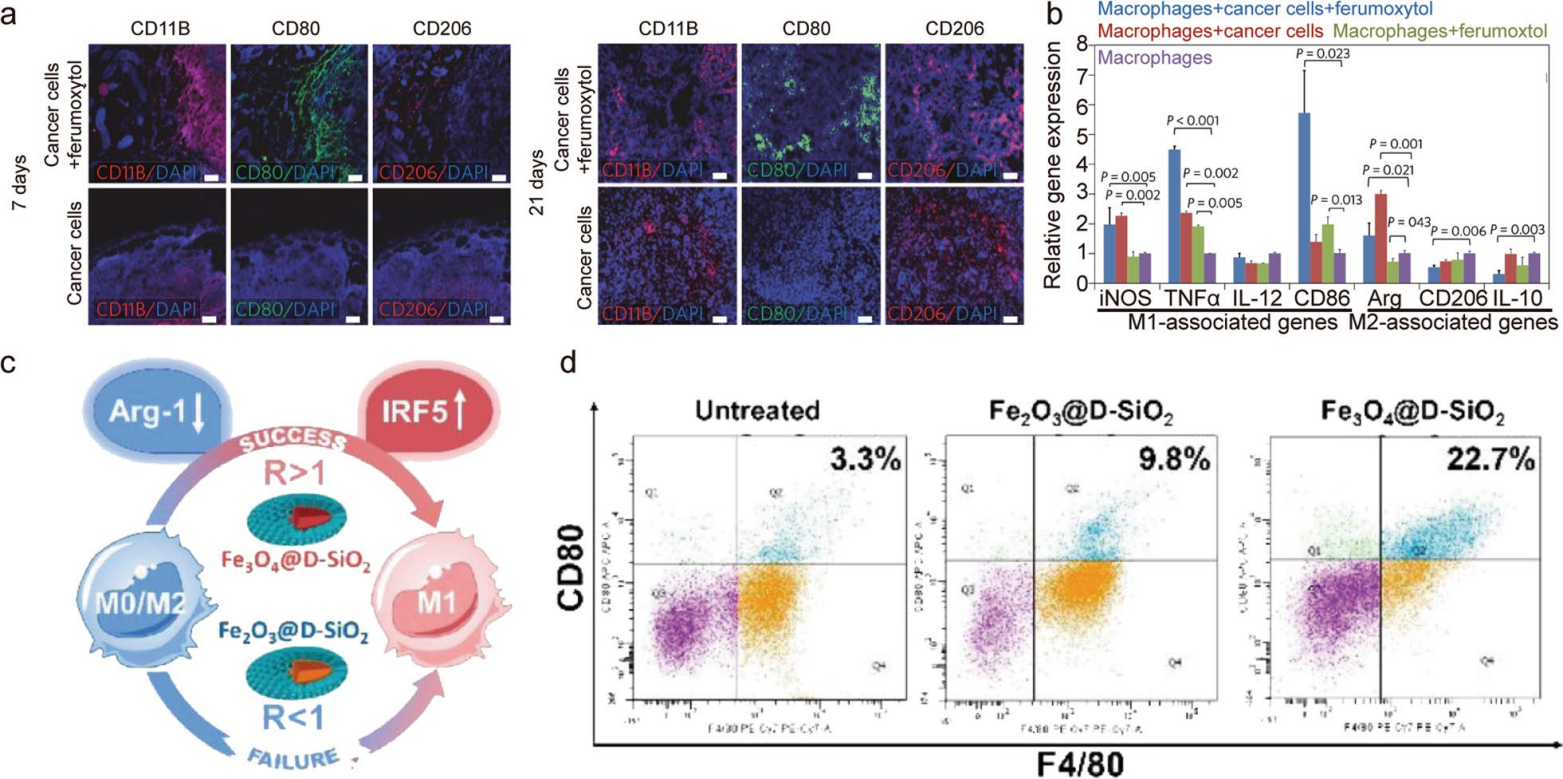
**a** Representative immunofluorescence staining for CD11b (red), CD206 (red), and CD80 (green) of MMTV-PyMT tumor sections obtained at 7 and 21 days; scale bars = 100 μm. **b** Co-cultures of cancer cells, macrophages, and ferumoxytol show signs of pro-inflammatory macrophage activation [95]. Copyright 2016, Springer Nature. c Mechanism of IONP-induced M1 activation. d Populations of M1 macrophages (labeled by F4/80+ and CD80+) in melanoma tumors treated with or without IONP@DSiO2 at day 12 after tumor implantation. All data are representative of at least three (n = 3) independent experiments for each experimental group and are displayed as mean ± SD; *P < 0.05 (Student’s t-test) [98]. Copyright 2019, American Chemical Society

Ferumoxytol NP was reported to suppress tumor growth by indirectly acting on the TME [95]; monocytes are recruited to the tumor site via local secretion of chemotactic cytokines, and, subsequently, are polarized to anti-inflammatory M2 macrophages. Previous studies had indicated that the amounts of iron-related proteins (*e.g.*, ferritin) in macrophages determine the functional polarization of macrophages [96]. High levels of ferritin and cathepsin L in M2 macrophages could convert them to M1 macrophages [97]. Ferumoxytol NPs promoted the up-expression of ferritin and cathepsin L in M2 macrophages, following a phenotypic shift towards M1 macrophages, as evidenced by the up-regulation of CD86 and TNF-α level [97]. M1-related TNFα and CD86 markers increased after inoculation with ferumoxytol plus cancer cells, whereas M2-related CD206 and interleukin (IL)-10 markers decreased (Fig. 6a, b). This M1 polarization leads to cancer cell apoptosis through the Fenton reaction. Persistent M1 polarization in turn can be induced by apoptotic cancer cells, which form a feedback loop to maintain TNFα and nitric oxide production.

Iron oxide nanoparticles (IONPs) can be labeled with large pore size silica to prevent aggregation and used for co-delivery applications [98]. The M1 phenotype was associated with iron-induced activation of the interferon regulatory factor 5 (IRF5) pathway without iNOS generation induced by the oxidative stress-related NF-κB pathway. Furthermore, Fe_3_O_4_ NPs suppressed M2 function by inhibiting the expression of arginase-1 (Arg-1). To demonstrate Fe_3_O_4_ NPs with superior ability for macrophage activation in vivo, both B16F10 cells and Fe_3_O_4_@D-SiO_2_ were administered into the right flank of C57BL/6 mice. It was observed that Fe_3_O_4_@D-SiO_2_ exhibited better antitumor efficacy than Fe_2_O_3_@D-SiO_2_, by inducing many more M1 macrophages within the TME (Fig. 6c, d).

### MNPs elicit a cytolytic T cell response

Wang et al*.* [99] studied the effect of Fe_3_O_4_ NPs on the mouse immune system. Mice were randomly divided into four groups and treated with saline or different doses of Fe_3_O_4_ NPs. Following intravenous injection of Fe_3_O_4_ NPs for 72 h, the induction of primary immune responses was examined by flow cytometry and enzyme-linked immunosorbent assay. There was no significant difference for the ratio of spleen to body weight amongst all groups. Furthermore, Fe_3_O_4_ NPs administered at the lowest dose exhibited higher lymphocyte transformation rates in spleen in comparison with the control group, while the proliferation of splenocytes was largely inhibited as the dose of Fe_3_O_4_ NPs increased. Mice treated with both low and medium doses of Fe_3_O_4_ NPs showed higher proportions of CD4^+^ and CD8^+^ T lymphocytes in peripheral blood, while there was no difference in the number of CD4^+^ T cells between low/medium-dose and high-dose groups. Interestingly, Fe_3_O_4_ NPs promoted the generation of IL-2, IFN-γ, and IL-10, but did not affect IL-4 generation in peripheral blood. The improved cytokine IL-2 and IFN-γ generation further increased the cytotoxic activity of CD8^+^ T lymphocytes [100, 101]. Thus, Fe_3_O_4_-MNPs influenced the immune functions of mice with a normal immune system in a dose-dependent manner.

## Immunotherapeutic activity of MNPs under a magnetic field

**Fig. 7:**
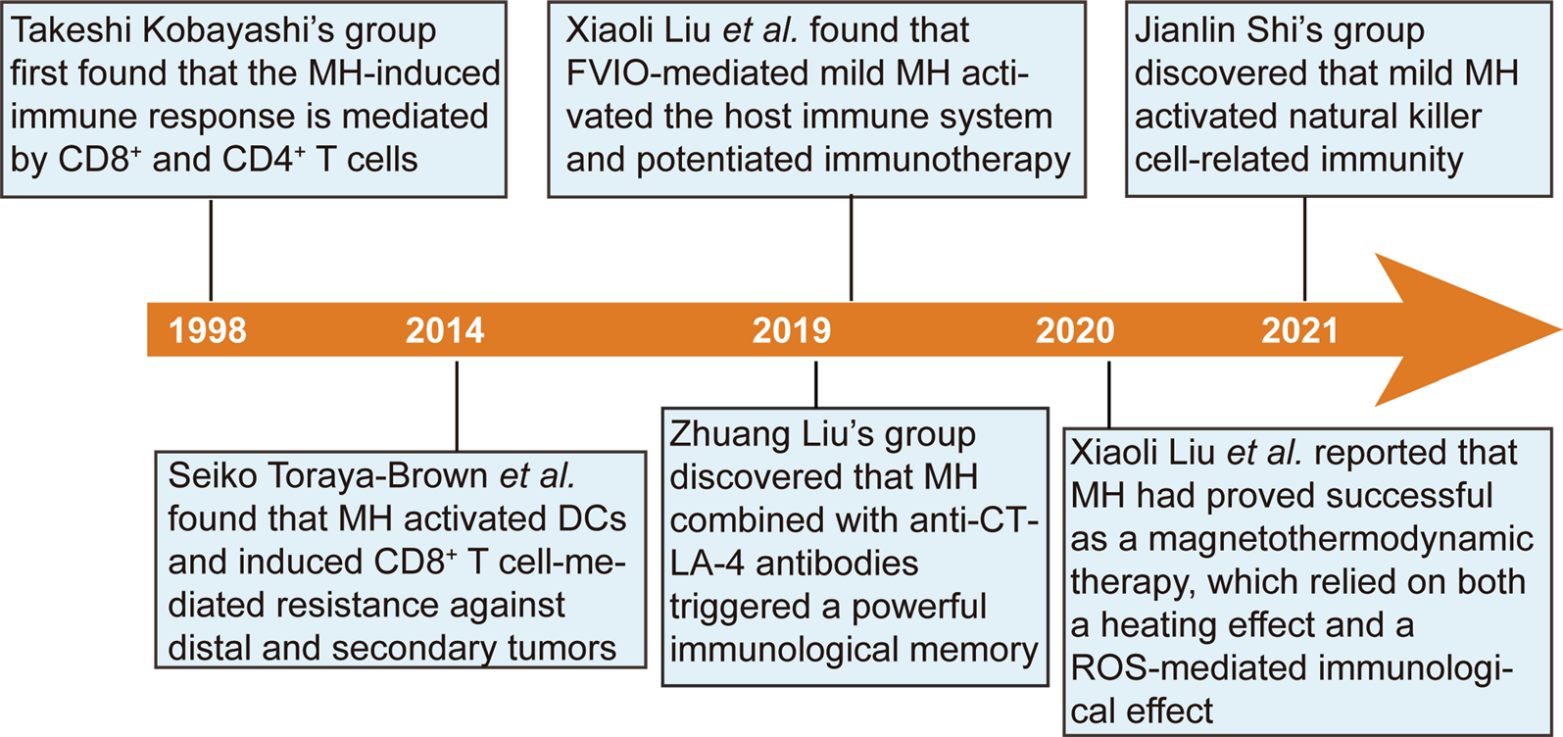
Major discoveries associated with the MH-mediated immune response

MNPs can produce various physicochemical effects in response to an external magnetic field. Under a medium-frequency alternating magnetic field (AMF; ~ 100–1000 kHz), MNPs can convert electromagnetic energy into heat for killing tumor cells or trigger drug release on-demand [102–105]. Magnetic hyperthermia (MH) is a physical treatment modality. AMF-actuated MH has transitioned into the clinic as a cancer therapy and is often used in combination with chemotherapy and radiotherapy to enhance its efficacy [106]. Multiple studies showed that MH therapy can improve antitumor immunity. MH can enhance antigen-specific T cell and/or natural killer (NK) cell responses [107] (Fig. 7), leading to strong immune memory and potentiation of immunotherapy.

**Fig. 8:**
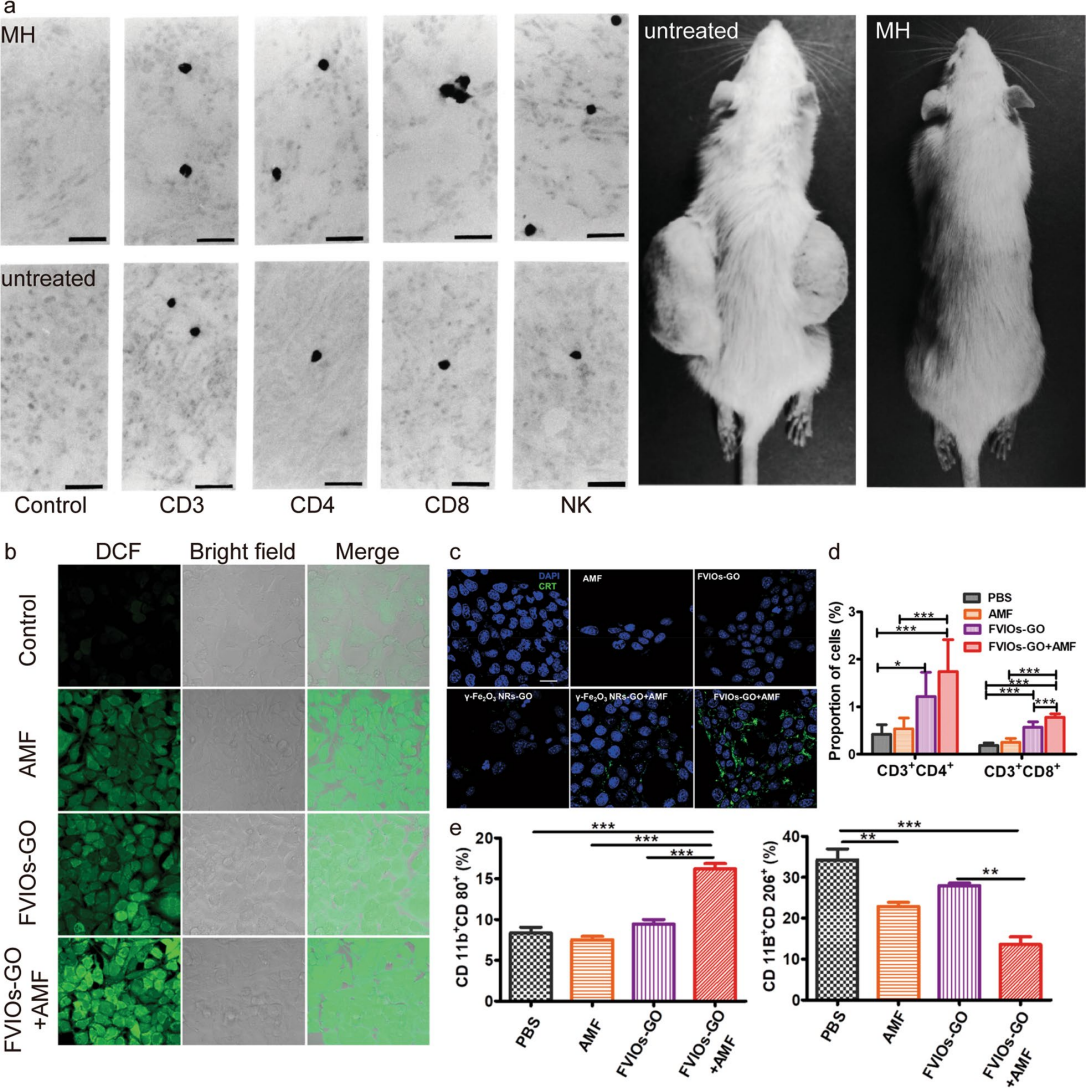
**a** Immunohistochemical staining for CD3^+^, CD4^+^, and CD8^+^ T cells, and NK cells; scale bar = 50 μm. Rats were photographed on day 28 [108]. Copyright 2005, John Wiley & Sons. **b** Confocal laser scanning microscopy images of ROS generation in 4T1 cells. **c** Confocal images showing CRT exposure on 4T1 tumor cells. **d** Percentages of tumor-infiltrating T cells in tumor tissue. **e** M1-polarization and M2-polarization phenotypes for different treatments. Data are reported as mean values ± SD; *0.01 < P < 0.05, **0.001 < P < 0.01, ***P < 0.001 [27]. Copyright 2020, American Chemical Society

MH was originally considered to exert merely a heating effect upon killing tumor cells at temperatures > 43 °C; temperature was the key index to assess the efficiency of tumor inhibition. The underlying mechanism of immunity activation by MH has not been elucidated. Pioneering work by the Kobayashi group [108] in 1998 revealed that MNP-mediated MH induced antitumor immunity not only through heating effects. CD8^+^ and CD4^+^ T cells were observed in T-9 glioma tumor tissues of rats with MH treatment, whereas no T cells were detected in this tissue without treatment (Fig. 8a). Moreover, the rats acquired long-lasting, T-9 cell-specific immunity. The activation of an immune response by MH was attributed to the release of HSPs to bind to and activate APCs; the T cell-mediated adaptive immune response was activated on receipt of the antigen presented by APCs. Liu et al*.*[27] subsequently demonstrated that MH is based on a synergistic combination of thermal and ROS-related immune effects to effectively eradicate tumors at a physiologically tolerable temperature. MH has been shown to exert a magnetothermodynamic (MTD) effect that is not limited to the macroscopic heating effect but could also exploit the immune effect associated with ROS. The authors designed a ferrimagnetic vortex-domain iron oxide nanoring and graphene oxide (FVIO-GO) with a high specific absorption rate (SAR) value and enhanced ROS production to meet the requisites for MTD therapy (Fig. 8b). FVIO-GO-mediated MTD elicited an ICD, as evidenced by the observation that 83% of 4T1 cells exposed CRT on the surface after MTD treatment, while only 37% of cells were detected after treatment by γ-Fe_2_O_3_ NPs plus AMF (the same SAR as FVIOs but negligible ROS generation) (Fig. 8c). These results clearly indicated that the amplified ROS generation was the critical factor in induction of ICD, which was therefore not limited to a heating effect. Flow cytometry results showed that FVIO-GO-mediated MTD promoted polarization of TAMs from the M2 to M1 phenotype (Fig. 8e), and increased T lymphocyte infiltration into the TME (Fig. 8d). Due to the combination effects, excellent in vivo antitumor efficacy was achieved at a low dosage and relatively shorter AMF exposure times.

In addition to the activation of the CTL-mediated immune response, a core − shell-structured Zn-CoFe_2_O_4_@Zn-MnFe_2_O_4_ was developed as an MH nanoagent [109] that exhibited excellent MH performance due to exchange-coupled magnetism between the core and shell as well as the presence of Zn^2+^ doping. Zn-CoFe_2_O_4_@Zn-MnFe_2_O_4_-mediated MH not only inhibited hepatocellular carcinoma cell proliferation and tumor growth, but also activated NK cells through up-regulating the expression of UL16-binding proteins and NK group II member D. Thus, the growth of both primary and metastatic HCC tumors was suppressed by MH-induced NK cell-mediated antitumor immunity in vivo.

MH has the ability to activate CD8^+^ T cells during the induction of an anti-tumor immune response, resulting in resistance against secondary tumor growth [110]. Based on this, it has been demonstrated that optimizing MH treatment (*e.g.,* temperature, duration, number of treatments) and combining with anti-CTLA-4 agents could generate long-lasting systemic immune responses that inhibit metastasis. A strong immune memory effect to resist tumor recurrence was also observed following the combination of MH with immunotherapy [111]. Improving ICD-associated immunogenicity induced by MH can also sensitize tumors to anti-PD-L1 immunotherapy. Liu et al*.* [28] reported that FVIO-mediated MH efficiently induced ICD and increased the percentage of CTL from 55.4% to 64.5% following combination with anti-PD-L1 agents. Additionally, combination therapy also suppressed the immunosuppressive response of the TME, as demonstrated by significant down-regulation of myeloid-derived suppressor cells. MH treatment activated the host immune system and cooperated with anti-PD-L1 immunotherapy to suppress potential metastatic proliferation as well as distant tumor growth.

## Conclusions and outlook

As discussed above, the functionalization of MNPs with immunotherapeutic agents for their effective delivery and the use of MNPs to generate ROS and MNP-mediated MH can improve immunotherapeutic activity, and further enhance anti-tumor efficacy. Unlike traditional clinic-based hyperthermia modalities in which hyperthermic action is directed at the tumor tissue level, MNP-mediated MH can deliver nano-heaters into cells and has the potential to effectively activate a series of intracellular stress responses, with the advantages of high selectivity, high precision, and low toxicity when triggering tumoricidal effects. With the increased importance of the immune system in cancer therapy, the potential of MNP-mediated MH in anti-tumor immunity is continuously being explored.

However, the design of MNPs and/or the optimization of the MH treatment with reasonably customized properties to achieve optimal immunological effects is crucial for the successful utilization of these nanotechnologies in cancer immunotherapy. Several challenges need to be overcome, especially for the clinical translation of MNP-based techniques into effective treatments, as considered below.

First, in vivo safety is one of the most important issues. Currently, amino silane-coated Fe_3_O_4_ NPs (NanoTherm^®^) are the only magnetic nanoagents approved and used in patients undergoing MH treatment. Evidence indicates that increasing intracellular ROS accelerates ICD induction efficacy, improving the tumor response to immunotherapy, resulting in a remarkable antitumor immune response. Therefore, optimization of the physical parameters (e.g., volume, composition, and morphology) of MNPs is needed to improve their magnetic responsive properties and Fenton catalytic activity to strengthen ROS generation. However, the release of bioactive free iron from MNPs has potential safety risks. Concerns remain about their toxicity on normal tissue when used in clinical practice. In addition, when designing optimal MNP-based delivery systems for hydrophobic immunotherapeutic agents, the efficacy, bioavailability, and off-target accumulation should be systematic evaluated.

Second, great progress has been made in enhancing immunogenicity and stimulating APCs to activate the immune system. Although artificially boosting the availability of TAAs and specific DAMPs by immunogenic MH efficiently transforms non-immunogenic forms to immunogenic forms, antitumor immune effects still require the involvement of tumor antigens–especially multiple neoantigens.

Third, various types of cells have unique roles in the TME. Once MNPs have accumulated in the tumor, MNPs can be internalized by tumor cells, T lymphocytes, DCs, macrophages, and MDSCs in the TME. The types of cells that phagocytose MNPs and their role need to be further investigated. A deep understanding of the manipulation mechanism of MNPs following cellular uptake should be prioritized. A comprehensive study of MNP–immune cell interactions in the presence or absence of AMF is necessary. Investigations of the effect of MNP-mediated hyperthermia on immunity-related cells, such as T lymphocytes, DCs, and MDSCs, in the TME are still in progress. Elucidation of the interactions between MNPs and/or MH and the immune system will provide theoretical evidence for clinical applications.

Fourth, among existing studies, combination therapy based on MNP-mediated MH and immune checkpoint blockade therapy has achieved satisfactory results; however, the synergistic mechanism needs to be further explored. Research should focus on the biological effects of immune cells under the action of MNPs plus AMF, including the direct regulation of immune checkpoints such as PD-1/PD-L1, CTLA-4, CD47, and inhibitory molecules.

Finally, further optimizing MH treatment (temperature, duration, and treatment times), and the proper time course for applying immunotherapies and MNP-based MH should be investigated. In addition, dosage is an important factor. The injection of higher quantities of MNPs may lead to an increased plasma iron concentration, which could induce oxidative stress and toxicities including cardiac and hepatic toxicity. In addition, chronic iron toxicity in patients with cirrhosis or hepatocellular carcinoma occurs after administration of high doses of iron (hepatic iron concentrations over 4 mg Fe/g liver wet weight). Thus, the combination of MNP-based MH with immunotherapy requires careful tuning.

The next generation of MH will be developed based on the broad understanding of complex immune regulation effects in vivo. The multifunctionality of MNP architecture needs to be further controlled to combine MNP-based MH and immunotherapy delivery in a single probe and to facilitate the administration of other treatment modalities including chemotherapy and radiotherapy, in parallel with MH. Solving these challenges should open the door for the broad clinical application of MNP-based MH in combination with immunotherapy.

## Data Availability

Data sharing is not applicable to this article as no datasets were generated or analyzed during the current study.
